# Fœtal Circulation

**Published:** 1855-07

**Authors:** 


*Fœtal Circulation.*—We insert the following, from the New Orleans Medical and Surgical Journal, as a pendant to the case of cyanosis in our Original Department:

Professor E. R. Peaslee, A.M., M.D., in an able monograph of 26 pages, on the Fœtal Circulation, sums up the results of his researches and conclusions, thus:—

*The View of the Fœtal Circulation required by the present state of Physiological Science.*—1. The human fœtus, during the last half of fœtal existence, has a *reptile* circulation—the mammal circulation commencing at birth; and the structure and the function of each particular part of its circulatory apparatus are in subservience to this fundamental fact. The characteristics of a reptile circulation are—I. The circulation of a *mixed* blood (and of the same degree of impurity) through both the aorta to the tissues, and through the pulmonary artery to the lungs; and II. The transmission of far less blood to the aërating apparatus than is sent through the aorta.

2. The foramen ovale with its valve is the only simple mechanism which could answer the requirements of the case, viz.: a temporary reptile circulation with a capability of instantaneous change to a permanent mammal circulation, the foramen becoming permanently closed about eight days after birth.

3. The ductus arteriosus is merely a "waste pipe," conducting into the nearest portion of the aorta that part of the blood sent into the trunk of the pulmonary artery, which the collapsed lungs of the fœtus are unable to receive. After birth the latter admit all the blood, and the ductus is, therefore, useless. It does not enter the descending aorta to avoid sending its blood to the head and upper extremities.

4. Though the lungs are more solid in the fœtus than after birth, they are probably permeated by about two-thirds of the blood entering the trunk of the pulmonary artery, and this is returned as *venous* blood to the left auricle.

5. The blood arriving in the right auricle from the two venæ cavæ, is completely intermixed by the diastole and systole of this cavity; and the same mixed blood is therefore transmitted through the foramen ovale into the left auricle. Or if by any possibility more placental blood enters that cavity, the venous blood, returned by the pulmonary veins, most probably counterbalances that advantage.

6. The eustachian valve cannot prevent the admixture of the blood from the venæ cavæ, nor direct that from the inferior cava at once through the foramen ovale; it merely prevents regurgitation from the auricle into the inferior vena cava, at the same time incidentally preventing the current from the superior cava from impinging so forcibly on that of the inferior. Hence, the valve of the foramen ovale replaces it to some extent, in respect to its principal function; and, therefore, it becomes atrophied in proportion as the latter is developed.

7. No artery in the body of the fœtus contains *arterial* blood. The aorta and pulmonary artery, and all their branches, contain a *mixed* blood, about five parts, at least, venous to one part placental. The precise proportions, however, are unimportant, the blood being of a *highly venous* character, and as impure in the aorta as in the pulmonary artery. Only the umbilical vein and the ductus venosus contain pure, aërated placental blood.

8. The umbilical arteries contain the same mixed blood as the aorta, and possibly return one-sixth of the blood received by that vessel; but this amount aërated in the placenta and returned by the umbilical veins, suffices to maintain the low standard of aëration in the fœtus.

9. The head and upper extremities of the fœtus do not receive a purer blood than the lower parts of the body. They, as well as the digestive and urinary apparatus, are early developed, in accordance with a general law of development.

10. The fœtal liver is a *depurating* organ only so far as it secretes bile, and, therefore, to a slight extent, though it does not thus convert venous into arterial blood. Its large development, from the placental blood abundantly distributed to it, has relation to its function as a *blood-making* and not a *bile-secreting* organ; and this blood becomes *venous* in the capillaries and the hepatic veins, as all analogy proves.

11. The trunk of the vena portæ is, in the fœtus, both the *nutrient artery* of the liver, and also corresponds to the vena portæ of the adult—its formative branches containing venous blood from which the bile in the meconium is probably secreted.

12. Anatomy, the history of development, and comparative physiology, combine to sustain the preceding propositions.

BOWDOIN COLLEGE, *April* 1, 1854.

